# Supplementary material for: Facile Preparation of Mechanically Robust and Functional Silica/Cellulose Nanofiber Gels Reinforced with Soluble Polysaccharides
Source: Nanomaterials (Basel). 2022 Mar 8;12(6):895. doi: 10.3390/nano12060895 (PMC8949125; doi:10.3390/nano12060895)
Supplement: Supplementary file 1 [file nanomaterials-12-00895-s001.zip › nanomaterials-1601700-supplementary.pdf]

Supporting Information

Facile Preparation of Mechanically Robust and Functional Silica/Cellulose Nanofiber Gels Reinforced with Soluble Polysaccharides

Marco Beaumont [1,*], Elisabeth Jahn [1], Andreas Mautner [2], Stefan Veigel [3], Stefan Böhmdorfer [1], Antje Potthast [1], Wolfgang Gindl-Altmutter [3] and Thomas Rosenau [2,4,*]

[1] Department of Chemistry, Institute of Chemistry of Renewable Resources, University of Natural Resources and Life Sciences Vienna, Konrad-Lorenz-Straße 24, 3430 Tulln, Austria; elisabeth.jahn@students.boku.ac.at (E.J.); stefan.boehmdorfer@boku.ac.at (S.B.); antje.potthast@boku.ac.at (A.P.)

[2] Faculty of Chemistry, Institute of Materials Chemistry and Research, Polymer and Composite Engineering (PaCE) Group, University of Vienna, Währinger Street 42, 1090 Vienna, Austria; andreas.mautner@univie.ac.at

[3] Department of Material Sciences and Process Engineering, Institute of Wood Technology and Renewable Materials, University of Natural Resources and Life Sciences Vienna, Konrad-Lorenz-Straße 24, 3430 Tulln, Austria; stefan.veigel@boku.ac.at (S.V.); wolfgang.gindl-altmutter@boku.ac.at (W.G.-A.)

[4] Johan Gadolin Process Chemistry Centre, Åbo Akademi University, Porthansgatan 3, FI-20500 Turku, Finland

* Correspondence: marcobeaumont1@gmail.com (M.B.); thomas.rosenau@boku.ac.at (T.R.)

Supporting experimental procedure

Bacterial cellulose (BC) cubes were obtained starting from commercial food product *nata de coco* from PT KOENG NUSANTARA ABADI, Indonesia. The cubes were washed extensively with deionized water to remove sugar and flavors. This process was repeated until the sweet odor was eliminated. Then the cubes were heated up to 90 °C in a 1% NaOH solution overnight (17 h) to remove the remaining proteins and then washed with water until neutral pH. Afterward, the bacterial cellulose was stored in deionized water at 8 °C.

Two BC cubes were immersed into a mixture of tetraethyl orthosilicate (TEOS, 2 mL, 1.0 g, 9.1 mmol), solvent (12 mL water to prepare BC-silica water samples, or a mixture of 10 mL EtOH and 2 mL water to yield BC–Silica water/EtOH samples) and HCl (0.16 mL of a 1% aqueous solution, 51 μmol). The samples were shaken for 30 h to complete TEOS hydrolysis, then 0.85 mL of a 0.1 mol/L NH_3 (85 μmol NH_3) was added. After gelation, 1 mL of the respective solvent was added and the samples were aged at 50 °C overnight in a closed container. Freeze-drying was conducted as reported in the main text. For supercritical drying, the samples were solvent exchanged to absolute EtOH (99.6% purity), and then transferred into a 300 mL autoclave equipped with a separator for carbon dioxide recycling (Separex, France). Drying was performed under a constant flow of $scCO_2$ (40 g min^{-1}) at 10 MPa and 40°C for 4.5 h. The system was then slowly and isothermally depressurized at a maximum rate of 0.1 MPa min^{-1}.

Table S1. Influence of different processing conditions with and without EtOH on the properties of the prepared bacterial cellulose (BC) gels. All samples were freeze-dried with prior solvent exchange to *t*BuOH. Compressive test curves for BC–silica samples are shown in Figure S1. The standard deviation of the average compressive strengths is reported ($n = 3$).

Sample	Density (g cm^{-3})	Specific surface area (m^2 g^{-1})	Porosity (%)	Compressive strength at 30% (kPa)
BC–Silica water	0.031	758	98.5	22 ± 4
BC–Silica water/EtOH (1:5)	0.028	897	98.6	13 ± 4
BC native	0.007	141	99.6	8 ± 5

Table S2. Influence of dying conditions on the properties of bacterial cellulose (BC) aerogels. Samples labeled with SC_ were dried via supercritical CO_2 drying and FD_ samples via lyophilization with prior solvent-exchange to *t*BuOH.

Sample	Density (g cm^{-3})	Specific surface area (m^2 g^{-1})	Porosity (%)
**SC**_BC-Silica water/EtOH	0.024	823	98.8
**FD**_BC-Silica water/EtOH	0.028	897	98.6
**SC**_BC native	0.004	206	99.7
**FD**_BC native	0.007	141	99.6

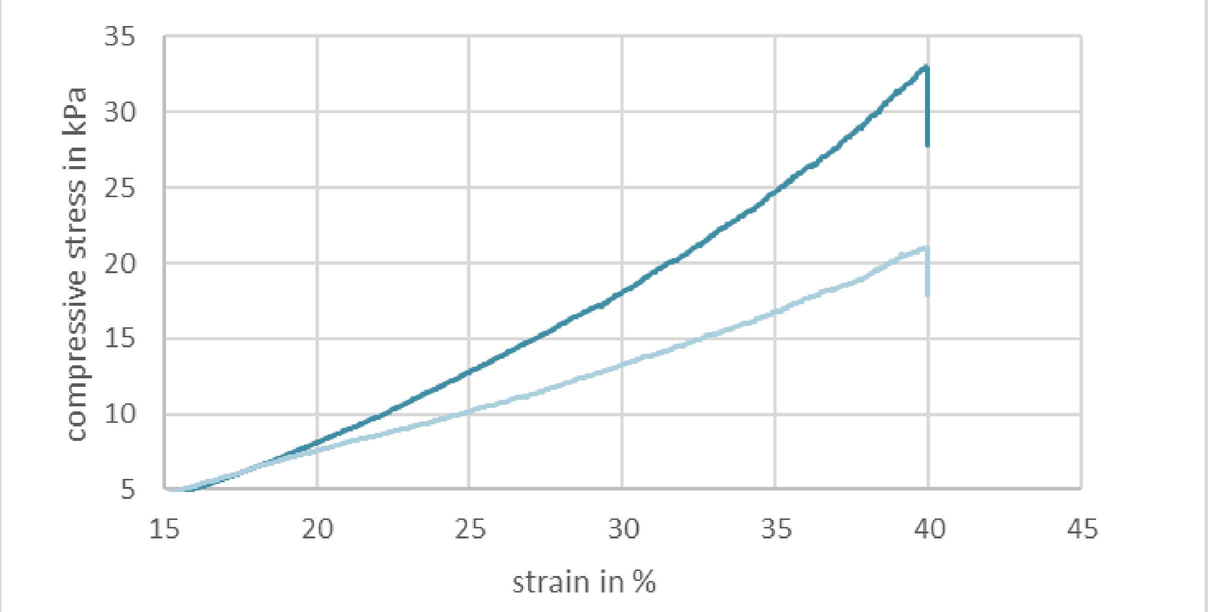

Figure S1. Compressive test of bacterical cellulose silica hydrogel samples prepared in EtOH/water (5:1, v:v, light blue line) and pure water (dark blue line). Measurements were conducted in wet conditions.

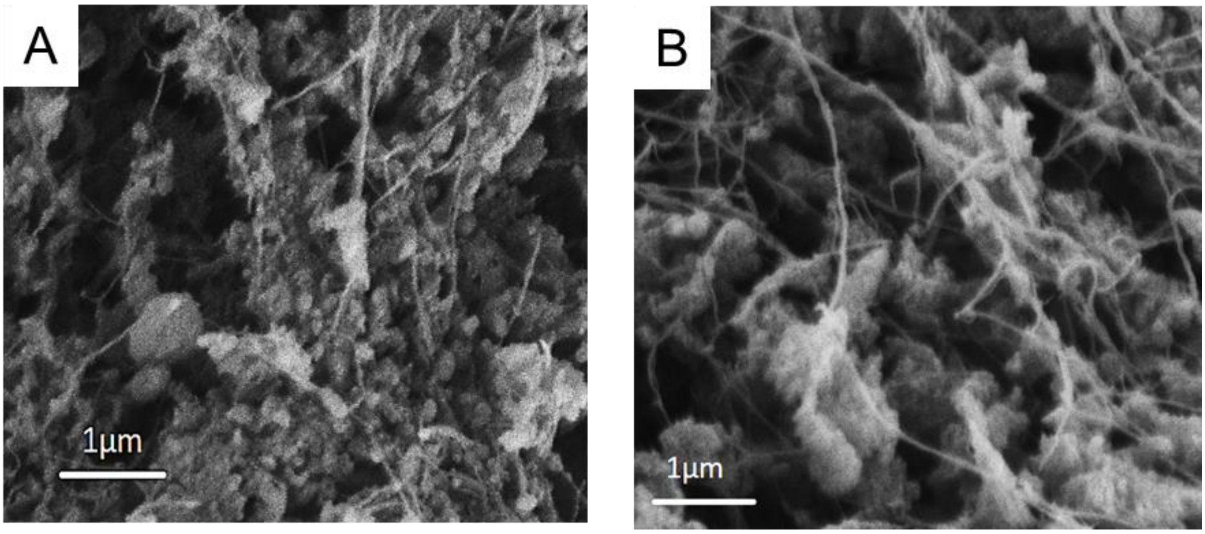

Figure S2: Scanning elecron micrographs of (**A**) the sample BC–Silica water/EtOH (1:5, v:v) in compairson to (**B**) BC–silica water. All samples were freeze-dried with prior solvent-exchange to *t*BuOH.
